# CT informs detection and treatment options in rheumatoid arthritis complicated by pulmonary non-tuberculous mycobacterial disease from the FIRST registry

**DOI:** 10.1136/rmdopen-2023-004049

**Published:** 2024-06-12

**Authors:** Masashi Funada, Yusuke Miyazaki, Shingo Nakayamada, Koshiro Sonomoto, Satoshi Kubo, Ippei Miyagawa, Hiroaki Tanaka, Yoshiya Tanaka

**Affiliations:** 1 The First Department of Internal Medicine, School of Medicine, University of Occupational and Environmental Health, Japan, Kitakyushu, Japan; 2 Department of Molecular Targeted Therapies (DMTT), University of Occupational and Environmental Health, Japan, Kitakyushu, Japan

**Keywords:** Arthritis, Rheumatoid, Risk Factors, Antirheumatic Agents

## Abstract

**Objective:**

To investigate the early detection of pulmonary non-tuberculous mycobacterial (PNTM) disease by CT before the initiation of molecular-targeted therapeutic drugs in patients with rheumatoid arthritis (RA) and the efficacy and safety of combined treatment with antibiotics.

**Methods:**

Patients with RA underwent chest CT before the introduction of molecular-targeted therapies in the Further Improvement of Rheumatoid arthritis Treatment registry. The primary endpoint was the number of patients who were detected by CT as having PNTM disease, complicating RA.

**Results:**

Of 4447 patients with RA who underwent chest CT, 107 had suspected PNTM disease, and 33 diagnoses were confirmed by culture. In 14 of the 33 patients, plain radiographs showed no abnormalities; PNTM disease was only observed on CT scans. The prevalence of PNTM disease in patients with RA requiring molecular-targeted treatment was six times higher than that in healthy individuals. 31 patients initiated molecular-targeted therapeutic drugs in combination with anti-NTM treatment, and 28 were followed up for 24 months. No significant difference was observed in the retention rate and RA disease activity at 24 months between the PNTM and non-PNTM groups. Coexisting PNTM disease did not affect treatment discontinuation. None of the 28 patients in the PNTM group experienced exacerbation of PNTM disease.

**Conclusion:**

CT screening before the initiation of molecular-targeted treatment enabled the detection of asymptomatic PNTM that was undetectable on plain radiographs. This study showed that molecular-targeted therapeutic drugs in combination with anti-NTM treatment could control the disease activity of both PNTM and RA.

**WHAT IS ALREADY KNOWN ON THIS TOPIC**

- The incidence of pulmonary non-tuberculous mycobacterial (PNTM) disease in patients with rheumatoid arthritis (RA) is higher than that in the general population. These patients tend to be resistant to conventional synthetic disease-modifying antirheumatic drugs; therefore, molecular-targeted treatment is often necessary while infectious lesions are controlled.

**WHAT THIS STUDY ADDS**

- In patients with RA, CT screening before the initiation of molecular-targeted therapeutic drugs can detect PNTM disease that is asymptomatic or undetectable on plain radiographs. The prevalence of PNTM disease is approximately six times higher in patients with RA requiring molecular-targeted treatment, which when combined with anti-NTM antibiotic use can control RA disease activity without PNTM relapse.

**HOW THIS STUDY MIGHT AFFECT RESEARCH, PRACTICE OR POLICY**

- The detection and evaluation of PNTM disease activity by CT screening contribute to the safe initiation of molecular-targeted treatment and controlling of both RA and PNTM disease activities.

## Introduction

Rheumatoid arthritis (RA) is a systemic inflammatory disease that causes progressive osteoarticular destruction and irreversible functional impairment.^1–3^ Since the advent of biological drugs targeting specific pathological molecules, the remission rate has improved dramatically.^4^ However, as patients age, complication rates also increase. Consequently, patient management before and during treatment is needed for patients’ safety.^5^ Regarding the extra-articular manifestations of RA, coexisting lung disease can affect prognosis. The incidence of bronchiectasis in patients with RA is extremely high at 40%,^6^ and bacterial bronchopneumonia secondary to bronchiectasis prevents the continuation of RA treatment, thereby reducing the survival rate.^7^

Bronchopneumonia includes pulmonary non-tuberculous mycobacterial (PNTM) disease; the prevalence of which has increased rapidly in recent years.^8^ The disease requires prolonged treatment, and many patients experience relapses.^9 10^ PNTM disease frequently complicates RA, with RA accounting for 8.7% of the underlying diseases of PNTM disease.^11^ The incidence of PNTM disease in patients with RA is twice as high as in those without RA. In patients with RA treated with anti-tumour necrosis factor (TNF) α inhibitors, the incidence is 10 times as high,^12^ and the use of TNFα inhibitors is a poor prognostic factor for RA complicated by PNTM disease.^13 14^ In this circumstance, initiation of molecular-targeted therapeutic drugs in patients with RA complicated by PNTM disease should be avoided. However, in reality, patients are resistant to conventional synthetic disease-modifying antirheumatic drugs (csDMARDs); therefore, molecular-targeted treatment is often necessary while infectious lesions are controlled.

However, the exact incidence of coexisting PNTM disease, including asymptomatic disease, in patients with RA needing molecular-targeted treatment is unknown. Moreover, it is still unknown whether molecular-targeted treatment can be continued and control the disease activity of RA in combination with PNTM treatment. This is a key question in clinical practice.

At our institution, for safer initiation of RA treatment, all patients who are initiating biological and targeted synthetic DMARDs (b/tsDMARDs) undergo non-contrast-enhanced CT, from the neck to the pelvis, to evaluate the risk of infections and malignant tumours.

In the current study, we performed chest CT screening before the initiation of molecular-targeted therapeutic drugs to accurately detect coexisting PNTM disease, including asymptomatic disease, to determine its incidence in patients with RA who were resistant to conventional treatment. Additionally, we investigated the retention rate of molecular-targeted therapeutic drugs and their effects on controlling the disease activity of RA when combined with PNTM treatment.

## Materials and methods

### Patients

Patients were recruited from the Further Improvement of Rheumatoid arthritis Treatment registry—a study of patients with RA receiving molecular targeted antirheumatic drugs at multiple institutions affiliated with our university hospital, the key station. The study was conducted by the University of Occupational and Environmental Health, Japan.^15–18^ RA was diagnosed when patients met either the 2010 American College of Rheumatology (ACR)/European League Against Rheumatism or the 1987 ACR classification criteria.^19 20^ The patients eligible for initiation of b/tsDMARDs were those who could not use csDMARDs, including methotrexate, or those in whom the joint symptoms were difficult to control with csDMARDs in standard doses or in combination with other b/tsDMARDs.

All patients who were enrolled in the FIRST registry from January 2005 to March 2023 underwent the screening test for PNTM disease. First, medical histories and physical examinations, including auscultation and plain chest radiography, were undertaken. Then, all patients underwent CT screening. Non-contrast CT scans from the neck to the pelvis were acquired using a 32-detector, 64-detector, 80-detector or 320-detector row CT scanner (online supplemental methods).

10.1136/rmdopen-2023-004049.supp1 Supplementary data

### Diagnosis of PNTM disease and initiation of anti-NTM drug treatment

When chest CT showed findings such as bronchiectasis, granular shadows, nodular shadows, infiltrative shadows and cavitation, PNTM disease was suspected based on the interpretation of radiologists, and sputum or bronchial lavage fluid was cultured. In addition, the finding of bronchiectasis, excluding traction bronchiectasis due to interstitial lung disease, on CT was determined based on the British Thoracic Society guidelines (online supplemental methods).^21^ Before or after the CT scans, PNTM disease was diagnosed according to the 2008 diagnostic criteria proposed by the Japanese Society for Tuberculosis and the Japanese Respiratory Society for patients who met the bacteriological criteria in addition to chest imaging findings (online supplemental methods).^22^ All patients with newly suspected PNTM disease on CT underwent sputum smearing and culturing. If the smear was negative, then bronchoscopy was performed. Bronchoscopy was also performed in smear-positive patients if the sputum culture was negative. In patients already diagnosed with PNTM disease, sputum smear and culture tests were performed after the confirmation of no worsening of the CT findings. If the smear was positive, a bronchoscopy was performed. A bronchoscopy was also performed for smear-negative patients at the treating physician’s discretion. If the above-mentioned bacteriologic examination was performed in another hospital immediately before the CT screening, we did not repeat it in our department.

Patients with newly diagnosed PNTM disease initiated anti-NTM treatment first. After confirmation that they were able to continue treatment, concomitant treatment with b/tsDMARDs was initiated. In patients who had already been diagnosed and were on treatment for PNTM disease, the absence of exacerbation of respiratory symptoms or an increase in pulmonary opacities was confirmed, and b/tsDMARDs were initiated while PNTM treatment continued. After initiation, patients were continuously followed up, with regular examinations by pulmonologists and plain radiography or CT. Specifically, PNTM disease monitoring after the initiation of b/tsDMARDs in this study was performed as follows. All patients underwent plain chest radiography at 6 and 12 months after the initiation and every 12 months thereafter. In patients in whom PNTM was suspected on CT screening before the initiation or whose condition was complicated by interstitial lung disease, follow-up CT was added every 6–12 months after initiation. Anti-NTM treatment, and subsequent RA treatment, were administered after we consulted pulmonologists, provided sufficient explanation to the patients and their families and obtained their consent. Exacerbation of PNTM after the initiation of b/tsDMARDs was defined as the appearance or exacerbation of respiratory symptoms due to PNTM disease or an increase in pulmonary opacities on images.

### Primary and secondary endpoints

The primary endpoint was the number of patients with RA complicated by PNTM disease that were detectable by CT screening before the initiation of b/tsDMARDs. The secondary endpoints were the clinical characteristics of the patients with coexisting PNTM disease, the retention rates of molecular-targeted therapeutic drugs 24 months after initiation of b/tsDMARDs in PNTM and non-PNTM groups, the RA disease activity (Clinical Disease Activity Index (CDAI)), the presence or absence of disease exacerbation in the PNTM group and the number and clinical characteristics of patients with new PNTM disease development after b/tsDMARDs initiation. Events leading to the discontinuation of b/tsDMARDs were analysed as adverse events.

### Statistical analysis

Patient characteristics are expressed as medians and IQRs or numbers (%). The last observation carried forward and non-responder imputation were also used to evaluate CDAI. Retention rates were assessed using the Kaplan-Meier method, and comparisons between groups were analysed using the log-rank test. The Student’s t-test and Mann-Whitney U test were used for between-group comparisons, and Pearson’s χ^2^ or Fisher’s exact test was used to compare categorical variables. To analyse the impact of coexisting PNTM disease on the continuation of molecular-targeted therapeutic drugs, the effects of background factors on the retention rate were analysed using a Cox proportional hazard model. Differences were considered statistically significant at the two-tailed p<0.05 level. All analyses were conducted by using JMP V.15.2 (SAS Institute).

## Results

### Study flow chart

**Figure 1:**
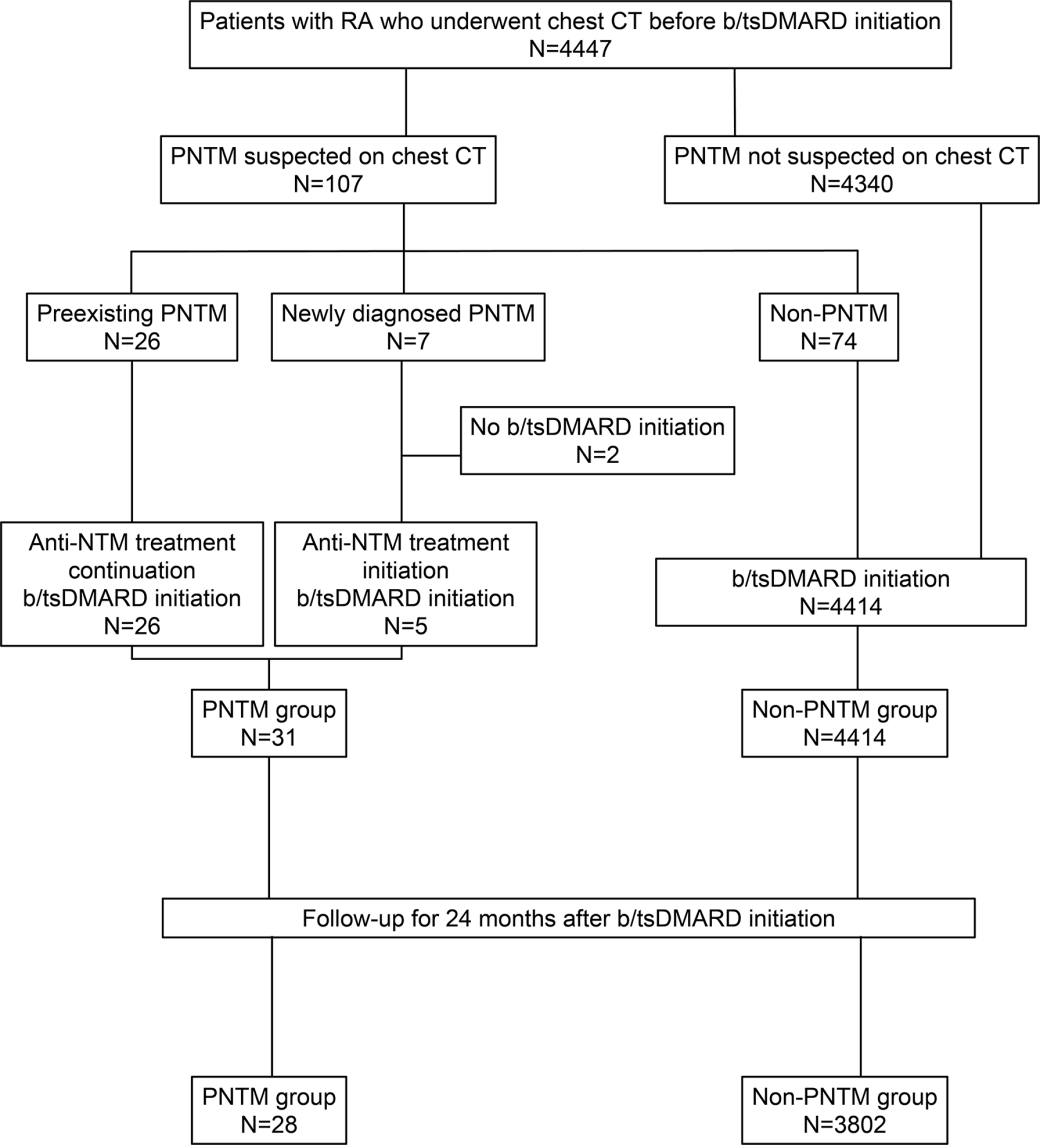
Patient selection process chest CT was performed in 4447 patients. Of the 107 patients with suspected pulmonary non-tuberculous mycobacterial (PNTM) disease, PNTM disease was observed in 33, including 7 with newly diagnosed PNTM disease and 26 with pre-existing PNTM disease. Biological and targeted synthetic disease-modifying antirheumatic drugs (b/tsDMARDs) were initiated in combination with anti-non-NTM treatment in 5 of the 7 patients with newly diagnosed PNTM disease and the 26 patients with preexisting PNTM disease. Of these patients, 28 with PNTM disease and 3802 patients without (non-PNTM group) were followed up for 24 months after initiation.

Figure 1 shows the flow chart of the 4447 patients with RA who underwent CT screening before initiation of b/tsDMARDs.

The following endpoints were analysed for each of the populations, as shown in figure 1. We evaluated the prevalence, clinical characteristics and risk factors of PNTM disease complication in RA and the PNTM detection rate of CT in 33 patients with detectable PNTM disease by CT screening and 4414 patients without PNTM disease (table 1, online supplemental tables 1 and 2, online supplemental figures 1 and 2). Of the 33 patients with PNTM disease, 31 initiated b/tsDMARDs and 2 did not initiate b/tsDMARDs. Of those two, one (online supplemental tables 3 and 4; case number 1) remained untreated for PNTM disease and was treated instead with escalating doses of methotrexate without worsening RA or PNTM disease while the other (case number 7) was diagnosed with concurrent chronic progressive pulmonary aspergillosis and PNTM disease for which anti-NTM treatment and antifungal agents were initiated. We also evaluated the breakdown of the initiated b/tsDMARDs, adverse events, retention rate, the effects of background factors on the retention rate, CDAI and the exacerbation of PNTM disease in 28 patients with PNTM disease and 3802 patients without PNTM disease who were continuously followed up for 24 months after treatment initiation.

10.1136/rmdopen-2023-004049.supp2 Supplementary data

10.1136/rmdopen-2023-004049.supp3 Supplementary data

**Table 1:** Patient characteristics between the PNTM and non-PNTM groups

	PNTM	Non-PNTM	P value
Number of cases	33	4414	
Female, n (%)	22 (66.7)	3544 (80.1)	0.041
Age (years)	68 (65–76)	63 (52–72)	0.001
BMI (kg/m^2^)	18.5 (17.2–22.4)	21.7 (19.5–24.3)	<0.001
Disease duration of RA (months)	149 (56–204)	60 (16–154)	0.001
RA stage I/II/III/IV (%)	10/30/33/27	23/43/18/16	0.120
28-tender joint count	7 (4–13)	6 (4–11)	0.488
28-swollen joint count	6 (3–10)	6 (4–8)	0.625
GH, VAS 0–100 mm	65 (40–80)	54 (35–73)	0.152
EGA, VAS 0–100 mm	50 (39–60)	45 (30–60)	0.212
Pain, VAS 0–100 mm	50 (34–73)	54 (33–74)	0.710
HAQ-DI	1.25 (0.88–2.50)	1.25 (0.63–1.87)	0.674
EQ-5D	0.60 (0.53–0.69)	0.59 (0.38–0.61)	0.210
CDAI	23.1 (18.6–32.0)	23.8 (16.2–33.3)	0.928
DAS28 (ESR)	5.74 (3.83–6.84)	5.54 (4.61–6.45)	0.173
CRP (mg/dL)	2.33 (1.05–4.92)	0.96 (0.20–3.12)	0.020
ESR (mm/hour)	73 (44–90)	46 (22–75)	0.078
RF positive n (%)	33 (100)	3465 (79.8)	<0.001
RF (IU/mL)	189 (50.3–627)	99.4 (43.7–221)	0.035
ACPA positive n (%)	33 (100)	3284 (74.4)	<0.001
ACPA (U/mL)	150 (56.8–730)	124 (36.1–428)	0.316
MMP-3 (mg/mL)	201 (95.8–337)	146 (68–310)	0.242
Brochiectasis n (%)	28 (84.8)	1276 (28.9)	<0.001
Interstitial lung diseases n (%)	7 (21.2)	310 (7.0)	<0.001
History of b/tsDMARD use, n (%)	22 (66.7)	1668 (37.8)	<0.001
History of TNFi use, n (%)	18 (81.8)	1422 (84.0)	0.663
History of IL-6Ri use, n (%)	7 (31.8)	422 (24.9)	0.483
History of ABT use, n (%)	13 (59.1)	370 (22.1)	<0.001
History of JAKi use, n (%)	4 (18.2)	124 (7.4)	0.006
MTX use, n (%)	21 (63.6)	3218 (72.9)	0.146
MTX dose (mg/week)	16 (10–16)	12 (8.0–16)	0.024
GC use, n (%)	11 (33.3)	1215 (27.5)	0.555
GC dose, mg/d, PSL equivalent	7.5 (3.5–23.0)	5.0 (2.5–7.5)	0.168
Radiographic features			
NB (%)	32 (97.0)		
NB+FC (%)	1 (3.0)		
NTM species			
*Mycobacterium avium* (%)	28 (84.8)		
*Mycobacterium intracellulare* (%)	2 (6.1)		
*Mycobacterium kansasii* (%)	3 (9.1)		

Data are medians (IQR) or numbers (%) of patients except where noted.

ABT, abatacept; ACPA, anti-citrullinated protein antibodies; BMI, body mass index; b/tsDMARD, biological and targeted synthetic disease-modifying antirheumatic drug; CDAI, Clinical Disease Activity Index; CRP, C reactive protein; DAS, Disease Activity Score; EGA, evaluator global assessment; EQ-5D, EuroQol 5 Dimension; ESR, erythrocyte sedimentation rate; FC, fibrocavitary disease ; GA, global assessment ; GC, glucocorticoid; HAQ-DI, Health Assessment Questionnaire Disability Activity Iindex; IL-6Ri, interleukin-6 receptor inhibitor; JAKi, Janus kinase inhibitor; MMP-3, matrix metalloproteinase 3; MTX, methotrexate; NB, nodular bronchiectatic disease; NTM, non-tuberculous mycobacteriosis; PNTM, pulmonary non-tuberculous mycobacterial; PSL, prednisolone; RA, rheumatoid arthritis; RF, rheumatoid factor; TNFi, tumour necrosis factor-alpha inhibitor; VAS, Visual Analogue Scale.

### Clinical characteristics of patients with RA complicated by PNTM disease

Based on chest CT findings, PNTM disease was suspected in 107 patients with RA and confirmed in 33 (0.74%). This yielded a prevalence rate of 742 per 100 000 persons.

Table 1 shows the clinical characteristics of the 33 patients with PNTM disease and 4414 patients without the disease (non-PNTM group). There was no difference in disease activity between the two groups. Regarding pre-existing lung lesions, the PNTM group had a significantly higher rate of bronchiectasis and interstitial lung disease complications. When the complication rates of bronchiectasis and interstitial lung disease were compared by dividing all patients by sex, the complication rates were significantly higher for male versus female patients: 33.3% (293/881) vs 28.3% (1009/3,566) (p=0.008) for bronchiectasis and 9.4% (83/881) vs 6.4% (227/3566) (p=0.003) for interstitial lung disease, respectively. The characteristics of the patients with PNTM disease were analysed using multiple logistic regression. Univariable analyses identified male sex, advanced age, low body mass index (BMI), high C reactive protein (CRP) level, positive rate for rheumatoid factor/anti-citrullinated protein antibodies (ACPA), presence of bronchiectasis and interstitial lung disease and history of b/tsDMARD use as influential factors. When multivariate analyses were performed with sex, age, BMI, bronchiectasis, interstitial lung disease and b/tsDMARDs used as confounding factors, which are reportedly associated with the development of PNTM disease or bacterial infection in patients with RA, the influential characteristics of patients with PNTM disease included male sex, advanced age, low BMI, the presence of bronchiectasis and interstitial lung disease, and b/tsDMARDs use (online supplemental table 1).^7 11 12 23–25^

### Characteristics of imaging findings in patients with RA complicated by PNTM disease

Online supplemental tables 2–4 show the characteristics of the pulmonary lesions in the 33 patients with coexisting PNTM disease. Regarding respiratory symptoms, mild cough and expectoration were observed in 5 (15.2%) patients while the other patients were asymptomatic. When the evaluation of pulmonary lesions using plain radiography alone (regular screening) was compared with evaluation using plain radiography and CT (CT screening), the PNTM disease detection rate was significantly higher for CT screening (CT screening vs regular screening: 33 (0.74%) vs 19 (0.43%) patients; p=0.039) (online supplemental figure 1). Abnormal shadows due to PNTM disease were observed on plain radiographs in 19 (57.6%) patients while the remaining 14 (42.4%) patients showed abnormal shadows only on CT scans. The isolated pathogens were *Mycobacterium avium* in 28 (84.8%) patients, *Mycobacterium intracellulare* in 2 (6.1%) and *Mycobacterium kansasii* in 3 (9.1%). According to the CT scan classification, 32 (97.0%) patients had the nodular bronchiectatic type and 1 (3.0%) had the nodular bronchiectatic plus fibrocavitary type (the cavitary lesions had been surgically resected).

Of the seven patients with newly diagnosed PNTM disease, five had no respiratory symptoms, and the remaining two only had minor coughs. Plain radiographs showed no abnormalities in six patients; however, nodular bronchiectasis was observed on CT scan (online supplemental figure 2). All five of the patients who were tested for serum anti-mycobacterium avium complex antibodies were positive. Diagnoses were confirmed by negative culture results from sputum specimens and positive culture results from bronchoalveolar lavage fluid specimens in five patients. Two patients had a history of b/tsDMARD use. Regarding the drug formulations used, one patient had received anti-TNFα inhibitors and interleukin (IL)-6 receptor inhibitors, and the other had received cytotoxic T-lymphocyte antigen 4-immunoglobulin (CTLA4-Ig).

### Efficacy and safety of molecular-targeted therapeutic drugs for patients with highly active RA complicated by PNTM disease

**Figure 2:**
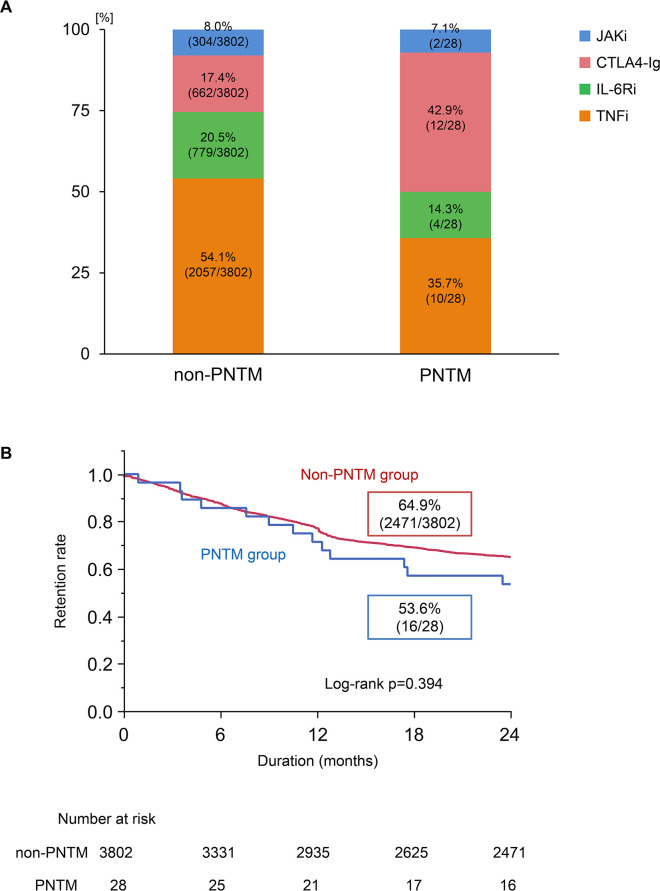
(A) Breakdown of biological and targeted synthetic disease-modifying antirheumatic drugs in the PNTM and non-PNTM groups. The highest initiation rate was observed for cytotoxic T-lymphocyte antigen 4-immunoglobulin (CTLA4-Ig) in the PNTM group and for TNFi in the non-PNTM group (Pearson’s χ^2^ test). (B) Retention rate of each b/tsDMARD initiated in the PNTM group and the non-PNTM group. No significant differences in the retention rates of b/tsDMARDs were observed between the PNTM and non-PNTM groups up to 24 months after the initiation of b/tsDMARDs. (log-rank test). b/tsDMARDs, biological and targeted synthetic disease-modifying antirheumatic drugs; JAKi, Janus kinase inhibitor; PNTM, pulmonary non-tuberculous mycobacterial.

Online supplemental table 5 shows the clinical characteristics of the 28 patients in the PNTM group and 3802 patients in the non-PNTM group who were continuously followed up for 24 months after treatment initiation. Figure 2A shows the breakdown of the initiated b/tsDMARDs by formulation type. Non-TNFα inhibitors were more likely to be initiated in the PNTM group than in the non-PNTM group (64.3% vs 45.9%, respectively; p=0.012). Anti-NTM antibiotic treatment was initiated or continued in all 28 patients, with 1 patient having a history of lobectomy for cavitary lesions (online supplemental table 2).

There was no significant difference in the retention rate of b/tsDMARDs at 24 months between the two groups (PNTM vs non-PNTM groups: 53.6% vs 64.9%; p=0.394; log-rank test) (figure 2B). All patients in the PNTM group continued antibiotic treatment during the follow-up period. Online supplemental figure 3 shows the clinical course of observation of b/tsDMARDs treatment in 33 patients with PNTM disease.

10.1136/rmdopen-2023-004049.supp4 Supplementary data

In the PNTM group, the 24-month retention rates of the molecular-targeted therapeutic drugs by formulation type were higher, at 75%, for CTLA4-Ig and anti-IL-6 receptor inhibitors than for anti-TNFα and Janus kinase inhibitors (p<0.001; log-rank test) (online supplemental figure 4). In the 28 patients in the PNTM group, the reasons for discontinuation of b/tsDMARDs prior to 24 months were inadequate responses in 9 patients (4 primary and 5 secondary non-responders), infection in 1 patient and patient requests in 2 patients. The patient who discontinued treatment because of infection developed *Pseudomonas aeruginosa* pneumonia during tofacitinib use (online supplemental table 6).

10.1136/rmdopen-2023-004049.supp5 Supplementary data

RA disease activity in the PNTM group had improved significantly 24 months after the initiation of b/tsDMARDs (baseline: 24 months=26.3±11.0:10.7±9.4; p<0.001) (online supplemental figure 5A). Remission was achieved 24 months after initiation of b/tsDMARDs in 14.8% of patients (online supplemental figure 5B).

10.1136/rmdopen-2023-004049.supp6 Supplementary data

**Figure 3:**
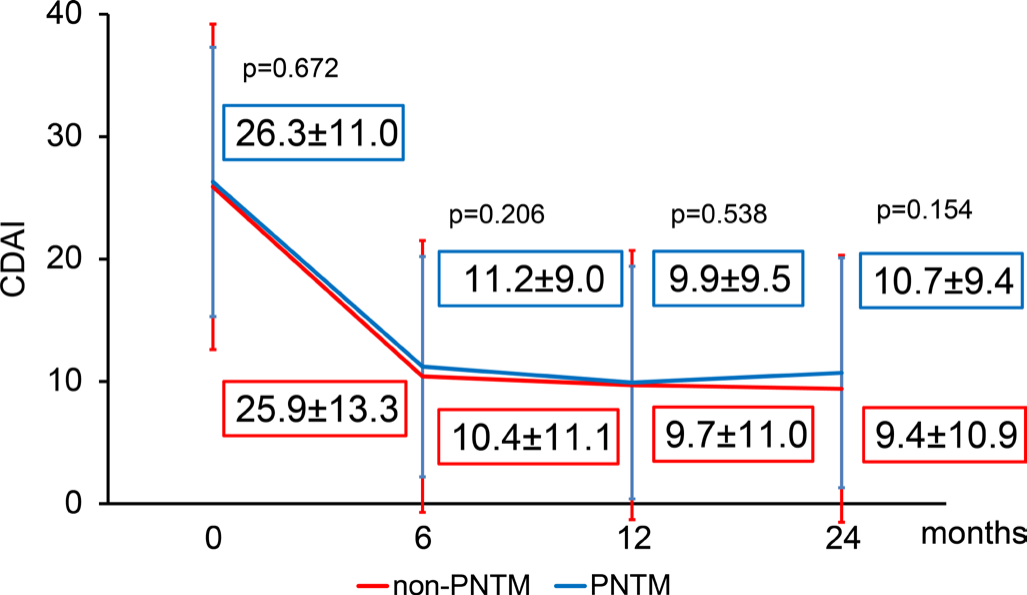
Clinical Disease Activity Index (CDAI) in the pulmonary non-tuberculous mycobacterial (PNTM) disease and non-PNTM groups. No significant differences were observed in the CDAI between the PNTM and non-PNTM groups up to 24 months after the initiation of biological and targeted synthetic disease-modifying antirheumatic drugs (Wilcoxon rank-sum test).

The CDAI 24 months after initiation of b/tsDMARDs was not significantly different between the PNTM and non-PNTM groups (10.7±9.4 vs 9.4±10.9, respectively; p=0.154) (figure 3).

### Effects of molecular-targeted therapeutic drugs on PNTM disease in patients with RA

None of the 31 patients with PNTM disease who initiated molecular-targeted treatment in combination with anti-NTM treatment experienced exacerbation of respiratory symptoms or imaging findings due to PNTM disease during the follow-up period. The pulmonary imaging findings improved in three patients after the initiation of b/tsDMARDs. All three patients initiated CTLA4-Ig and had no respiratory symptoms at the time of initiation. After initiation, they achieved low RA disease activity, which was maintained for 24 months or longer.

### Impact of PNTM disease on the retention rate of molecular-targeted therapeutic drugs in patients with highly active RA

A Cox proportional hazard model was used to examine whether coexisting PNTM affected the number of consecutive days treated with b/tsDMARDs (table 2). The univariable analysis showed no impact of coexisting PNTM disease on the number of consecutive days treated with b/tsDMARDs (HR 1.24; 95% CI 0.76 to 2.02; p=0.396). The impact of coexisting PNTM disease on discontinuation of b/tsDMARDs was adjusted for sex, age, duration of RA, the Health Assessment Questionnaire Disability Activity Index, CDAI, CRP, ACPA positive rate, complication of bronchiectasis and interstitial lung disease, history of b/tsDMARD use, history of methotrexate use, history of glucocorticoid use and initiation of new treatment with anti-TNFα inhibitors, and multivariable analysis of the impact was performed. Even after adjustment for these factors, coexisting PNTM disease did not affect the number of consecutive days treated with b/tsDMARDs (HR 1.30; 95% CI 0.78 to 2.19; p=0.315).

**Table 2:** HR for risk of b/tsDMARD discontinuation in all patients with RA (n=3830): Cox regression

	Univariable analysis			Multivariable analysis		
	HR	95% CI	P value	HR	95% CI	P value
Male/female	1.11	0.99 to 1.24	0.064	1.13	0.98 to 1.30	0.085
Age	0.99	0.99 to 1.00	<0.001	0.99	0.99 to 0.99	0.016
BMI	0.99	0.98 to 1.01	0.364			
Disease duration	1.00	1.00 to 1.00	0.005	1.00	0.99 to 1.00	0.047
HAQ-DI	1.04	0.99 to 1.10	0.133	1.09	1.01 to 1.18	0.037
EQ-5D	0.84	0.62 to 1.15	0.273			
CDAI	1.00	1.00 to 1.00	0.768	0.99	0.99 to 1.01	0.354
DAS28 (ESR)	1.02	0.78 to 1.43	0.876			
CRP	1.01	0.99 to 1.01	0.387	1.00	0.98 to 1.02	0.882
ESR	1.00	1.00 to 1.00	0.447			
RF positive	0.92	0.83 to 1.03	0.158			
ACPA positive	0.96	0.85 to 1.09	0.534	0.99	0.87 to 1.11	0.828
MMP-3	1.00	1.00 to 1.00	0.510			
Comorbidities						
Bronchiectasis	1.17	1.06 to 1.28	0.001	1.24	1.10 to 1.41	0.001
Interstitial lung diseases	0.94	0.79 to 1.13	0.539	1.04	0.83 to 1.30	0.743
PNTM	1.24	0.76 to 2.02	0.396	1.30	0.78 to 2.19	0.315
Drug use history						
b/tsDMARD	1.00	0.91 to 1.10	0.969	1.25	1.11 to 1.41	<0.001
MTX	1.18	1.06 to 1.31	0.003	1.05	0.91 to 1.21	0.480
GC	1.00	0.90 to 1.10	0.971	1.11	0.96 to 1.27	0.150
Initiated drug						
TNFi	1.63	1.48 to 1.80	<0.001	1.85	1.64 to 2.08	<0.001
IL-6Ri	0.71	0.63 to 0.81	<0.001			
ABT	0.87	0.76 to 0.98	0.025			
JAKi	0.42	0.33 to 0.54	<0.001			

ABT, abatacept; ACPA, anti-citrullinated protein antibodies; BMI, body mass index; b/tsDMARD, biological and targeted synthetic disease-modifying antirheumatic drug; CDAI, Clinical Disease Activity Index; CRP, C reactive protein; DAS, Disease Activity Score; EQ-5D, EuroQol 5 Dimension; ESR, erythrocyte sedimentation rate; GC, glucocorticoid; HAQ-DI, Health Assessment Questionnaire Disability Activity Index; IL-6Ri, interleukin-6 receptor inhibitor; JAKi, Janus kinase inhibitor; MMP-3, matrix metalloproteinase 3; MTX, methotrexate; NTM, non-tuberculous mycobacteriosis; PNTM, pulmonary non-tuberculous mycobacterial; RA, rheumatoid arthritis; RF, rheumatoid factor; TNFi, tumour necrosis factor-alpha inhibitor.

Similarly, a Cox proportional hazard model was used to investigate background factors that affected the number of consecutive days treated with b/tsDMARDs (table 3). Low scores on the EuroQol 5 Dimension (HR 0.02; 95% CI 0.00 to 0.75; p=0.036) and initiation of b/tsDMARDs other than CTLA4-Ig (HR 0.24; 95% CI 0.07 to 0.84; p=0.012) were associated with fewer consecutive days treated with b/tsDMARDs.

**Table 3:** HR for risk of b/tsDMARD discontinuation in PNTM group (n=28): univariable Cox regression

	Univariable analysis		
	HR	95% CI	P value
Male/female	8.39	2.82 to 25.0	<0.001
Age	0.94	0.87 to 1.01	0.107
BMI	1.17	0.99 to 1.38	0.066
Disease duration	0.99	0.99 to 1.00	0.061
HAQ-DI	1.16	0.62 to 2.13	0.643
EQ-5D	0.02	0.00 to 0.75	0.036
CDAI	1.01	0.96 to 1.04	0.760
DAS28 (ESR)	1.13	0.76 to 1.85	0.586
CRP	0.92	0.77 to 1.03	0.213
ESR	0.99	0.98 to 1.01	0.288
MMP-3	1.00	1.00 to 1.01	0.179
Comorbidities			
Bronchiectasis	3.12	0.41 to 23.78	0.272
Interstitial lung diseases	3.17	1.11 to 9.04	0.031
Drug use history			
b/tsDMARD	1.62	0.55 to 4.75	0.379
MTX	2.22	0.62 to 7.87	0.219
GC	1.16	0.41 to 3.27	0.776
Initiated drug			
TNFi	2.73	0.98 to 7.64	0.055
IL-6Ri	0.69	0.16 to 3.07	0.627
ABT	0.24	0.07 to 0.84	0.012

ABT, abatacept; BMI, body mass index; b/tsDMARD, biological and targeted synthetic disease-modifying antirheumatic drug; CDAI, Clinical Disease Activity Index; CRP, C reactive protein; DAS, Disease Activity Score; EQ-5D, EuroQol 5 Dimension; ESR, erythrocyte sedimentation rate; GC, glucocorticoid; HAQ-DI, Health Assessment Questionnaire Disability Activity Index; IL-6Ri, interleukin-6 receptor inhibitor; MMP-3, matrix metalloproteinase 3; MTX, methotrexate; NTM, non-tuberculous mycobacteriosis; PNTM, pulmonary non-tuberculous mycobacterial; RA, rheumatoid arthritis; RF, rheumatoid factor; TNFi, tumour necrosis factor-alpha inhibitor.

### New PNTM disease development after initiation of molecular targeted therapeutic drugs

Examination of the incidence of PNTM disease after the initiation of b/tsDMARDs in this study revealed that, of the 4414 RA patients without PNTM complications, 3 of the 3802 patients followed up to 24 months after the initiation of b/tsDMARDs developed PNTM disease for an incidence of 39.5/100 000 person-years. Two of the three patients with PNTM disease were suspected of having the condition on CT screenings but did not meet the diagnostic criteria. Another 74 patients who were suspected of having PNTM disease on CT screening but did not meet the diagnostic criteria were routinely screened for the condition using plain chest radiography and CT. Of the 62 patients followed up to 24 months after the initiation of b/tsDMARDs, 2 developed PNTM disease as described above (incidence: 1612.9/100 000 person-years). However, the remaining patient had interstitial lung disease and was diagnosed and treated at an early disease stage after undergoing regular CT screenings. Of the 3740 patients who were not suspected of having PNTM disease on CT screenings (n=4340) and followed up to 24 months, only this patient developed the condition for an incidence of 13.4/100 000 person-years.

Online supplemental table 7 shows the clinical characteristics of the three patients who developed PNTM disease after the initiation of b/tsDMARDs. In all three cases, the b/tsDMARDs were discontinued, oral antimicrobial therapy was started, CTLA4-Ig was subsequently initiated and the RA disease activity improved.

## Discussion

The study examined the following possibilities: (1) It can be suggested that the prevalence of PNTM disease is higher in patients with RA who require molecular-targeted treatment than in the general population and that of our predictions. (2) Asymptomatic PNTM disease, undetectable on plain radiographs, can be detected by CT screening before the initiation of molecular-targeted treatment. (3) In patients with RA complicated by PNTM disease, anti-NTM treatment may allow the continuation of molecular-targeted therapeutic drugs and control of RA disease activity without exacerbation of PNTM disease, similar to patients with RA not complicated by PNTM disease.

To date, there have been no studies involving patients with highly active RA requiring molecular-targeted treatment in which CT was performed in all patients to examine the accurate incidence of coexisting PNTM disease before treatment initiation. In this study, most of the seven patients with newly diagnosed PNTM were asymptomatic and their plain radiographs showed no abnormal shadows. Thus, if CT had not been performed, PNTM disease might not have been diagnosed until symptom onset. CT screening allows the detection of asymptomatic PNTM disease with normal radiographs and the initiation of anti-NTM treatment before the initiation of b/tsDMARDs. The Japanese diagnostic criteria for PNTM disease, unlike the European and American criteria, allow diagnosis of asymptomatic PNTM disease.^22 26^ This is based on the high participation rates for CT scans and medical checkups in Japan, which allow diagnosis before the onset of symptoms.

However, CT scans are associated with a concern about the development of malignant tumours due to the probabilistic effect of exposure to radiation. However, we have performed CT screening before the initiation of b/tsDMARDs and detected malignant tumours at a high rate: 1 in 66 patients. This indicates that the detection rate of CT screening exceeds the incidence of malignant tumours due to exposure to radiation from CT scans (1 in 200 individuals).^27–30^ Additionally, the mean effective dose for CT in this registry study was 14 mSv, which was lower than that in preceding studies. Thus, the effect of CT scans on the development of malignant tumours appears smaller. Furthermore, the current study showed that CT screening is effective for the detection of malignant tumours and PNTM disease and the evaluation of disease activity. The benefits of CT screening appear to outweigh the risk of developing malignant tumours after exposure to radiation from CT scans.

In the current study, the prevalence of PNTM disease in patients with RA requiring molecular-targeted treatment was 742 per 100 000 persons. The prevalence of PNTM disease in the general population in Japan is 112–129 per 100 000 persons, based on mortality statistics and the analysis of data from medical check-ups.^31 32^ Thus, PNTM disease prevalence in patients with highly active RA requiring molecular-targeted treatment is approximately six times higher than that in the general population.

The characteristics of patients with RA complicated by PNTM disease included male sex, advanced age, low BMI, prevalence of bronchiectasis and interstitial lung disease, and history of b/tsDMARD use. Advanced age and low BMI have been reported to be risk factors for PNTM disease without RA^24^ while low BMI (<18.5 kg/m^2^) has also been reported to be a risk factor for RA.^25^ Regarding sex, although a study involving the general population showed that female sex was a risk factor for PNTM disease,^11^ the current study identified male sex as a risk factor. In patients with RA, male sex is a risk factor for interstitial lung disease.^33^ In this study, male patients had a significantly higher frequency of interstitial lung disease and bronchiectasis complications than female patients.

We initiated b/tsDMARDs in combination with anti-NTM treatment in 31 of 33 patients with PNTM disease and followed 28 patients for 24 months. None of the 28 patients exhibited PNTM disease exacerbation. Additionally, RA in nine of these patients responded inadequately to b/tsDMARDs. All nine patients continued anti-NTM treatment after changes in b/tsDMARDs, and no exacerbation of PNTM disease was observed in the subsequent course. As revealed by the current study, patients with asymptomatic mild PNTM disease and those with the nodular bronchiectatic type stabilised by treatment can continue b/tsDMARDs, without exacerbation of PNTM disease, while continuing anti-NTM treatment.

No significant differences were observed in the retention rate of molecular-targeted therapeutic drugs at 24 months or the control of RA disease activity between the PNTM and non-PNTM groups. To date, there have been no studies comparing the retention rate of b/tsDMARDs nor RA disease activity between patients with RA complicated/not complicated by PNTM disease with the former on anti-NTM treatment. The current study showed that anti-NTM treatment combined with b/tsDMARDs could control RA disease activity to a similar extent to that in patients with RA not complicated by PNTM disease. Additionally, the multivariable analysis showed that coexisting PNTM disease did not affect the discontinuation of b/tsDMARDs. Among the drug formulations initiated, CTLA4-Ig, which is recommended for patients with coexisting PNTM disease by the guidelines of the ACR,^34^ was associated with a high initiation rate and the highest retention rate in the PNTM group. When background factors associated with the discontinuation of b/tsDMARDs in the PNTM group were analysed with a Cox proportional hazard model, the univariable analysis identified the initiation of CTLA4-Ig as a factor associated with treatment continuation. CTLA4-Ig improved joint symptoms of RA while some patients who continued CTLA4-Ig for 24 months or longer showed improved PNTM imaging findings. Therefore, the treatment retention rate can remain high by initiating CTLA4-Ig for RA complicated by PNTM disease.

The incidence of PNTM in patients with RA initiated on biological agents is reportedly 105.0–328.1/100 000 person-years.^12 35 36^ These results suggest the following: (1) the incidence of PNTM tends to be higher in patients with CT-suspected PNTM disease even after the initiation of b/tsDMARDs and (2) the CT screenings performed in this study detected asymptomatic and early-onset PNTM disease, which may have reduced its incidence after the initiation of b/tsDMARDs compared with previous studies. In fact, in our department, CT scans were performed every 6–12 months after the initiation of b/tsDMARDs in patients with suspected PNTM disease and complicated interstitial lung disease, enabling an early diagnosis. Therefore, if PNTM disease is suspected on screening CT prior to the initiation of b/tsDMARDs, follow-up CT imaging should be continued after the initiation. On the other hand, if such CT findings are absent, it is highly likely that the patients would not develop PNTM disease after the initiation of b/tsDMARDs.

The study has some limitations. First, because of the small number of patients with PNTM disease, when the retention rates in patients with RA complicated by PNTM disease were calculated according to drug formulations, characteristics differed among patients treated with different drug formulations, which might have contributed to the difference in the retention rates. CTLA4-Ig showed a high retention rate, although many patients treated with this formulation had a history of b/tsDMARD use, which is a factor contributing to the discontinuation of b/tsDMARDs. Moreover, most patients with PNTM disease in the current study were asymptomatic or had symptoms that were stabilised by treatment. Thus, the results of the current study cannot be applied to RA complicated by highly active PNTM disease. It is necessary to individualise treatment, such as prioritisation of enhanced treatment of PNTM disease. The risk of developing PNTM disease is assumed to be much lower in healthy subjects than in patients with RA in this study in terms of preexisting lung disease and induction medications. On the other hand, if CT screenings were performed on healthy subjects, the prevalence may be higher, but this is only speculation due to a lack of CT screenings of healthy subjects. In addition, after the initiation of b/tsDMARDs, standardised CT follow-up is not performed in patients without suspected PNTM disease or interstitial lung disease. Although the results of this study suggest a low incidence of PNTM disease in these patients, its diagnosis may be delayed due to a patient’s asymptomatic status and minor imaging abnormalities. Just as standardised CT follow-up is performed only in certain patients, bacteriological examination is also performed only in patients with abnormal chest imaging findings or respiratory symptoms after initiation of b/tsDMARDs, which may underestimate the incidence of PNTM disease after initiation of b/tsDMARDs.

In this study, we performed CT screening in patients with RA requiring molecular-targeted treatment and were able to detect PNTM disease that was asymptomatic or undetectable on plain radiographs. We found that the prevalence of PNTM disease was approximately six times higher in patients with RA requiring molecular-targeted treatment than in healthy individuals. The detection and evaluation of PNTM disease activity by CT screening contributed to the safe initiation of molecular-targeted treatment. Additionally, b/tsDMARDs could be continued without exacerbation of PNTM disease with combined anti-NTM treatment, and the retention rate of molecular-targeted therapeutic drugs and control of RA disease activity were also comparable between patients with and without PNTM disease. PNTM disease is chronic and progressive, and long-term observational studies are necessary to evaluate the safety of b/tsDMARDs. In the future, studies with a longer observation period and a larger sample size are needed.

## Data Availability

Data are available on reasonable request.
