# Supplementary material for: CT informs detection and treatment options in rheumatoid arthritis complicated by pulmonary non-tuberculous mycobacterial disease from the FIRST registry
Source: RMD Open. 2024 Jun 12;10(2):e004049. doi: 10.1136/rmdopen-2023-004049 (PMC11177696; doi:10.1136/rmdopen-2023-004049)
Supplement: Supplementary data [file rmdopen-2023-004049supp001.pdf]

SUPPLEMENTARY MATERIAL

SUPPLEMENTARY METHODS

CT scanning technique

The CT scanning technique utilized collimation of either 0.5 or 1 mm, a rotation time of 0.5 s, and 120 kVp. Automatic tube current modulation was used, specifically z-axis modulation with the Real E.C. technique, manufactured by CANON MEDICAL SYSTEMS CORPORATION in Tochigi, Japan. The noise level was set to 10 S.D. to ensure optimal image quality while keeping radiation exposure at a minimum. The dose length product measured 950 mGy cm.

Bacteriological criteria for PNTM diagnosis (1)

PNTM was diagnosed based on any of the following criteria: 1. two or more positive cultures from different sputum specimens; 2. one or more positive cultures from bronchial lavage fluid specimens; 3. one or more positive cultures from tissue, bronchial lavage fluid, or sputum specimens in addition to histological findings consistent with *Mycobacterium* infection in patients undergoing transbronchial lung biopsy or lung biopsy; or 4. identification of two or more positive cultures of bacterial species and specialists' views in patients infected with rare bacterial species or bacterial species frequently isolated from the environment.

(1) Kurashima A. [Japanese new guidelines for nontuberculous mycobacterial pulmonary disease]. Kekkaku 2010;85:87–93. Japanese.

CT features of bronchiectasis (2)

Bronchiectasis is defined by bronchial dilatation as suggested by one or more of the following: 1) Bronchoarterial ratio >1 (internal airway lumen vs adjacent pulmonary artery); 2) Lack of tapering; 3) Airway visibility within 1cm of costal pleural surface or touching mediastinal pleura.

(2) Hill AT, Sullivan AL, Chalmers JD, et al. British Thoracic Society Guideline for bronchiectasis in adults. Thorax. 2019 Jan;74(Suppl 1):1-69.

Supplementary Table 1 Characteristics of patients with RA complicated by pulmonary non-tuberculous mycobacterial disease (PNTM)

	Univariable Analysis			Multivariable Analysis		
	Odds ratio	95% CI	*P*-Value	Odds ratio	95% CI	*P*-Value
Male	2.17	1.01–4.66	0.046	2.84	1.20–6.69	0.017
Age	1.05	1.02–1.09	<0.001	1.04	1.01–1.08	0.049
BMI	0.77	0.69–0.86	<0.001	0.75	0.67–0.85	<0.001
Disease duration of RA	1.00	0.99–1.00	0.065			
28-tender joint count	0.99	0.94–1.05	0.743			
28-swollen joint count	0.99	0.92–1.06	0.704			
GH, VAS 0–100 mm	1.01	0.99–1.02	0.272			
EGA, VAS 0–100 mm	1.01	0.99–1.02	0.465			
Pain, VAS 0–100 mm	1.00	0.99–1.01	0.989			
HAQ-DI	1.10	0.72–1.68	0.673			
EQ-5D	0.22	0.03–1.67	0.155			
CDAI	1.00	0.98–1.03	0.867			
DAS28 (ESR)	1.09	0.91–1.31	0.344			
CRP	1.11	1.04–1.19	0.003			
ESR	1.01	1.00–1.02	0.084			
RF positive (>45)	3.45	1.32–9.02	0.005			
ACPA positive (>13.5)	3.98	1.20–13.1	0.007			
MMP-3	1.00	0.99–1.00	0.826			
Prevalence of bronchiectasis	14.79	5.12–42.74	<0.001	9.89	3.36–29.09	<0.001

Prevalence of interstitial lung disease	4.56	1.92–10.83	0.006	3.26	1.30–8.15	0.021
Prior use of biologics	2.67	1.28–5.57	0.009	2.47	1.13–5.43	0.024
MTX use at baseline	0.66	0.31–1.41	0.296			
GC use at baseline	1.54	0.73–3.25	0.266			

ACPA: anti-citrullinated protein antibodies; BMI: body mass index; b/tsDMARD: biologic and targeted synthetic disease-modifying antirheumatic drug; CDAI: clinical disease activity index; CI: confidence interval; CRP: C-reactive protein; DAS: disease activity score; EGA VAS: evaluator global assessment of disease activity visual analogue scale; EQ-5D: EuroQol 5 Dimension; ESR: erythrocyte sedimentation rate; GC: glucocorticoid; GH VAS: patient's global assessment of disease activity visual analogue scale; HAQ-DI: health assessment questionnaire disability activity index; MMP-3: matrix metalloproteinase 3; MTX: methotrexate; NTM: non tuberculous mycobacteriosis; RA: rheumatoid arthritis; RF: rheumatoid factor.

Supplementary Table 2 Clinical characteristics of pulmonary non-tuberculous mycobacterial disease (PNTM) in patients with rheumatoid arthritis (RA)

Variables	N=33
Respiratory symptoms, n, (%)	5 (15.2)
Anti-MAC antibody positive, n, (%)	8 (24.2)
Abnormal chest plain radiograph, n (%)	19 (57.6)
Co-existing bronchiectasis, n (%)	28 (84.8)
Co-existing ILD, n (%)	7 (21.2)
Computed tomography (CT) patterns, n, (%)	
NB	32 (97.0)
NB + FC	1 (3.0)
Bacterial species, n (%)	
M. avium	28 (84.8)
M. intracellulare	2 (6.1)
M. kansasii	3 (9.1)
Anti-NTM treatment initiation, n, (%)	
RFP/EB/CAM	23 (69.7)
INH/EB/RFP	3 (9.1)
RFP/LVFX/CAM	1 (3.0)
RFP/EB/MFLX	1 (3.0)

EB/CAM	3 (9.1)
Lobectomy + RFP/CAM	1 (3.0)
No treatment	1 (3.0)

Data are medians (IQRs: interquartile ranges) except where noted. CAM: clarithromycin; EB: ethambutol; FC: fibrocavitary disease; ILD: interstitial lung disease; INH: isoniazid; LVFX: levofloxacin; MAC: mycobacterium avium complex; MFLX: moxifloxacin; NA: not available; NB: nodular bronchiectatic disease; NTM: non-tuberculous mycobacteriosis; RFP: rifampicin.

Supplementary Table 3 Clinical characteristics of rheumatoid arthritis (RA) in each patient with pulmonary non-tuberculous mycobacterial disease (PNTM) and concurrent RA

Case number	Age	BMI (kg/m^2)	Stage	Disease duration (m)	RF/ACPA	CDAI/ DAS28 (ESR)	Prior use of biologics	MTX (mg/w)	PSL (mg/day)	Initiation b/tsDMARDs
1	70s	17.3	IV	288	43.5/26.9	34.0/5.64	-	10	0	-
2	60s	18.0	III	144	50.3/34.0	23.0/5.84	+	10	0	ABT
3	80s	15.3	I	4	246.1/235.6	16.0/5.66	-	0	0	ABT
4	70s	23.6	IV	204	43.1/257.2	23.1/6.12	+	16	0	ETN
5	60s	19.4	II	132	188.7/402.9	22.3/5.80	-	0	0	ABT
6	50s	19.1	III	192	403.8/2113.2	19.9/5.76	-	0	0	-
7	50s	23.5	IV	216	710/108.1	32.6/6.38	-	6	3	IFX
8	80s	20.4	IV	180	113.3/6.6	16.5/4.95	+	0	0.5	ETN
9	60s	16.1	IV	264	221.6/11.4	36.4/6.70	-	16	0	ABT
10	70s	20.4	III	252	80.0/12.5	23.7/5.70	-	0	0	ABT
11	60s	20.1	III	72	18.1/52.3	9.9/1.75	-	0	20	ABT
12	60s	15.4	III	73	57.2/54.8	26.0/6.21	+	14	5	ETN
13	60s	13.6	IV	308	1304/71	69.0/7.86	+	0	15	TCZ
14	60s	23.8	II	21	91.9/85.1	22.2/5.14	+	16	0	TOF
15	60s	17.8	III	159	38/89.5	32.3/6.12	+	8	0	ETN
16	70s	17.2	II	36	27/92.4	29.8/6.32	-	16	0	ETN
17	60s	20.4	II	73	311.3/103.4	30.1/4.66	+	6	0	ABT
18	60s	24.5	I	29	63.5/109.9	13.5/3.56	+	16	0	TCZ
19	70s	18.5	IV	120	87.5/111.4	21.5/5.68	+	6	0	ABT
20	70s	17.8	IV	492	195.7/117	18.6/5.16	+	16	0	ABT
21	60s	23.8	I	18	300.5/292.6	28.7/5.69	-	16	0	ADA
22	70s	18.3	II	83	1828/448.7	24.0/5.47	+	0	10	TOF
23	70s	18.0	III	48	175.2/461.8	20.8/5.31	-	12	0	TCZ
24	60s	14.0	IV	202	143.3/819.5	36.5/4.89	+	16	2	CZP

25	60s	14.3	III	195	221.4/797.1	36.7/6.83	+	16	0	ETN
26	70s	22.4	II	48	48.7/932.5	24.3/5.67	+	16	0	ABT
27	70s	18.0	II	48	307.9/1028	24.7/7.13	+	0	4	ABT
28	70s	18.5	II	45	1120/1385	37.0/5.97	+	0	0	ABT
29	70s	16.7	II	199	315.5/3551	20.3/6.07	+	0	0	TCZ
30	50s	23.8	III	144	783.3/100	40.0/7.75	+	12	7.5	ETN
31	60s	18.2	III	149	121.1/78.4	23.0/5.84	+	14	8	TCZ
32	70s	20.9	III	202	185.5/162.2	18.6/4.42	+	0	7	ABT
33	60s	19.0	II	112	222.3/150.1	22.0/5.54	+	16	0	TCZ

ABT: abatacept; ACPA: anti-citrullinated protein antibodies; ADA: adalimumab; BMI: body mass index; b/tsDMARD: biologic and targeted synthetic disease-modifying antirheumatic drug; CDAI: clinical disease activity index; CZP: certolizumab pegol; DAS: disease activity score; ESR: erythrocyte sedimentation rate; ETN: etanercept; IFX: infliximab; MTX: methotrexate; PSL: prednisolone; RF: rheumatoid factor; TCZ: tocilizumab; TOF: tofacitinib.

Supplementary Table 4 Clinical characteristics of pulmonary non-tuberculous mycobacterial disease in each patient with rheumatoid arthritis

Case number	Respiratory symptoms	Bacterial species	acid-fast bacilli smear (sputum)	Sputum culture	Bronchial lavage fluid culture	Anti-MAC antibody positive*	Abnormal chest plain radiograph	CT patterns	Co-existing bronchiectasis	Co-existing ILD	Anti-NTM treatment
1	-	*M. avium*	+	+	NA	NA	-	NB	+	-	-
2	-	*M. kansasii*	-	-	+	NA	-	NB	+	-	INH/EB/RFP
3	-	*M. avium*	+	+	NA	+	-	NB	+	-	RFP/EB/CAM
4	+：mild cough	*M. avium*	-	-	+	+	-	NB	+	-	RFP/EB/CAM
5	-	*M. avium*	-	-	+	+	+	NB	+	-	RFP/EB/CAM
6	+：mild cough	*M. avium*	-	-	+	+	-	NB	+	+	RFP/EB/CAM
7	-	*M. avium*	-	-	+	+	-	NB	+	-	RFP/EB/CAM
8	-	*M. avium*	-	-	-	NA	+	NB	+	-	RFP/EB/CAM
9	-	*M. intracellulare*	-	+	-	+	+	NB	+	-	RFP/LVFX/CAM
10	-	*M. avium*	-	-	NA	-	-	NB	+	-	RFP/EB/CAM
11	-	*M. avium*	-	-	-	NA	+	NB	-	+	RFP/EB/CAM
12	+：mild cough	*M. avium*	-	-	-	NA	+	NB	-	+	RFP/EB/CAM

13	+：mild cough	*M. avium*	-	-	NA	-	+	NB + FC	+	+	RFP/EB/ MFLX + lobectomy
14	-	*M. avium*	-	-	-	+	+	NB	+	-	RFP/EB/ CAM
15	-	*M. kansasii*	-	-	NA	-	-	NB	+	-	INH/EB/ RFP
16	-	*M. avium*	-	-	-	NA	+	NB	+	-	RFP/EB/ CAM
17	-	*M. avium*	-	-	NA	NA	+	NB	+	-	EB/CAM
18	-	*M. avium*	-	-	NA	NA	+	NB	+	-	RFP/EB/ CAM
19	-	*M. avium*	-	-	NA	NA	+	NB	+	-	RFP/EB/ CAM
20	-	*M. avium*	-	-	NA	NA	-	NB	+	-	RFP/EB/ CAM
21	-	*M. avium*	-	-	NA	NA	+	NB	+	-	RFP/EB/ CAM
22	-	*M. avium*	-	-	NA	-	+	NB	-	+	EB/CAM
23	-	*M. intracellulare*	-	-	-	+	+	NB	+	-	RFP/CAM
24	-	*M. avium*	-	-	NA	-	-	NB	+	-	RFP/EB/ CAM
25	-	*M. avium*	-	-	NA	-	-	NB	+	-	RFP/EB/ CAM
26		*M. avium*	NA	NA	NA	NA	-	NB	+	-	RFP/EB/ CAM
27	-	*M. avium*	-	-	NA	-	+	NB	+	-	RFP/EB/ CAM
28	-	*M. avium*	-	-	NA	-	+	NB	-	+	RFP/EB/ CAM
29	+：mild cough	*M. avium*	-	-	NA	-	-	NB	-	+	EB/CAM
30	-	*M. kansasii*	-	-	NA	-	+	NB	+	-	INH/EB/ RFP
31	-	*M. avium*	-	-	-	-	+	NB	+	-	RFP/EB/ CAM

32	-	*M. avium*	-	-	-	-	+	NB	+	-	RFP/EB/CAM
33	-	*M. avium*	-	-	-	-	-	NB	+	-	RFP/EB/CAM

CAM: clarithromycin; CT: computed tomography; EB: ethambutol; FC: fibrocavitary; ILD: interstitial lung disease; INH: isoniazid; LVFX: levofloxacin; MAC: mycobacterium avium complex; MFLX: moxifloxacin; NA: not available; NB: nodular branchiectatic; NTM: non tuberculous mycobacteriosis; RFP: rifampicin.

* Anti-MAC antibody is synonymous with the anti-glycopeptide-core IgA antibody.

Supplementary Table 5 Patient characteristics in the PNTM and non-PNTM groups

	PNTM	Non-PNTM	*P*-Value
Number of cases	28	3,802	
Female n (%)	19 (67.9)	3,091 (81.3)	0.070
Age (years)	68 (63–77)	63 (52–71)	0.001
BMI (kg/m^2)	18.4 (16.8–21.9)	21.7 (19.5–24.2)	<0.001
Disease duration of RA (M)	126 (48–201)	60 (15–149)	0.014
RA stage I/II/III/IV (%)	10/30/33/27	22/44/18/16	0.120
28-tender joint count	8 (5–12)	8 (4–13)	0.777
28-swollen joint count	6 (5–8)	6 (4–10)	0.955
GH, VAS 0–100 mm	58 (40–80)	54 (35–74)	0.325
EGA, VAS 0–100 mm	48 (34–58)	45 (30–60)	0.513
Pain, VAS 0–100 mm	50 (43–66)	54 (33–74)	0.868
HAQ-DI	1.25 (0.66–2.13)	1.25 (0.66–2.00)	0.611
EQ-5D	0.59 (0.46–0.73)	0.60 (0.51–0.69)	0.220
CDAI	23.7 (20.8–32.3)	24.0 (16.2–33.6)	0.672
DAS28 (ESR)	5.82 (5.35–6.37)	5.56 (4.70–6.49)	0.176
CRP (mg/dL)	2.07 (0.70–5.20)	1.04 (0.22–3.20)	0.039
ESR (mm/h)	65 (37–82)	46 (23–75)	0.096
RF positive n (%)	28 (100)	3,002 (78.9)	0.007
RF (IU/mL)	159 (58.8–309)	99.0 (44.1–220)	0.072
ACPA positive n (%)	28 (100)	2,286 (75.2)	0.001
ACPA (U/mL)	111 (42.4–507)	76.8 (12.2–323)	0.293
MMP-3 (mg/mL)	201 (88.6–356)	147 (69–315)	0.234
Bronchiectasis n (%)	23 (82.1)	1,115 (29.3)	<0.001
Interstitial lung diseases n (%)	6 (21.4)	284 (7.5)	0.002
History of b/tsDMARD use n (%)	17 (60.7)	1,366 (35.9)	0.007
History of TNFi use n (%)	12(70.6)	1,158(85.0)	0.098
History of IL-6R-anti use n (%)	3(17.7)	297(21.7)	0.689
History of ABT use n (%)	9(52.9)	263(19.3)	<0.001
History of JAKi use n (%)	4(25.5)	47(3.4)	0.003
MTX use n (%)	19 (67.9)	2,860 (75.2)	0.369
MTX dose (mg/week)	16 (8.0–16)	10 (8.0–16)	0.084
GC use n (%)	11 (39.3)	1,038 (27.3)	0.157
GC dose (PSL mg/day)	5.0 (2.0–15.0)	5.0 (2.5–7.5)	0.486
Radiographic features			
NB (%)	27 (96.4)		
NB+FC (%)	1 (3.6)		
NTM species			
M. avium (%)	23 (82.1)		
M. intracellurare (%)	2 (7.1)		
M. kansasii (%)	3 (10.8)		

Data are medians (IQRs: interquartile ranges) except where noted. ABT: abatacept; ACPA: anti-citrullinated protein antibodies; BMI: body mass index; b/tsDMARD: biologic and targeted synthetic disease-modifying antirheumatic drug; CDAI: clinical disease activity index; CRP: C-reactive protein; DAS: disease activity score; EGA VAS: evaluator global assessment of disease activity visual analogue scale; EQ-5D: EuroQol 5 Dimension; ESR: erythrocyte sedimentation rate; FC: fibrocavitary disease; GC: glucocorticoid; GH VAS:

patient's global assessment of disease activity visual analogue scale; HAQ-DI: health assessment questionnaire disability activity index; IL-6R-anti: interleukin-6 receptor inhibitor; JAKi: janus kinase inhibitor; MMP-3: matrix metalloproteinase 3; MTX: methotrexate; NB: nodular bronchiectatic disease; NTM: non-tuberculous mycobacteriosis; PSL: prednisolone; RA: rheumatoid arthritis; RF: rheumatoid factor; TNFi: tumour necrosis factor-alpha inhibitor.

Supplementary Table 6 Events leading to discontinuation of b/tsDMARDs in the PNTM group

Events leading to discontinuation of b/tsDMARDs	n=12
Inadequate response, n, (%)	9 (32.1)
Withdrawal of consent for treatment, n, (%)	2 (7.2)
Bacterial pneumonia*	1 (3.6)

b/tsDMARD: biologic and targeted synthetic disease-modifying antirheumatic drug.
*The cause of death was pseudomonas aeruginosa pneumonia.

Supplementary Table 7 Clinical characteristics of each patient who developed pulmonary non-tuberculous mycobacterial disease after initiation of b/tsDMARDs

Age	BMI (kg/m^2)	Suspicion of PNTM on CT before b/tsDMARDs initiation	Initiation b/tsDMARDs	Duration of b/tsDMARDs administration until PNTM onset (m)	Respiratory symptoms	Bacterial species	Sputum culture	Bronchial lavage fluid culture	CT patterns	Co-existing BE	Co-existing ILD	Anti-NTM treatment
70s	21.4	+	TCZ	13	-	*M. avium*	-	+	NB	+	-	RFP/EB/CAM
70s	23.3	-	CZP	17	Mild cough	*M. avium*	-	+	NB	-	+	EB/CAM
60s	13.3	+	CZP	18	-	*M. avium*	-	+	NB	+	-	RFP/EB/CAM

BE: bronchiectasis; BMI: body mass index; b/tsDMARD: biologic and targeted synthetic disease-modifying antirheumatic drug; CAM: clarithromycin; CT: computed tomography; CZP: certolizumab pegol; EB: ethambutol; ILD: interstitial lung disease; NB: nodular branchiectatic; NTM: non tuberculous mycobacteriosis; RFP: rifampicin; TCZ: tocilizumab.

SUPPLEMENTARY FIGURE LEGENDS

Supplementary Figure 1. Detection rates of pulmonary non-tuberculous mycobacterial (PNTM) disease by computed tomography (CT) and regular screening
The detection rate of PNTM disease by CT screening is significantly higher than that by regular screening (Pearson's chi-square test).

Supplementary Figure 2. Imaging findings in six cases with no abnormality on plain radiographs
Plain chest radiographs show no abnormal shadows in the lung fields, whereas chest computed tomography (CT) shows bronchiectasis, patchy shadows, and granular shadows (arrows).

Supplementary Figure 3. Clinical course of observation of biologic and targeted synthetic disease-modifying antirheumatic drug (b/tsDMARD) treatment in 33 patients with pulmonary non-tuberculous mycobacterial (PNTM) disease
Case number 1 through 7 represent newly diagnosed PNTM disease. We did not initiate b/tsDMARD treatment for case numbers 1 and 6 but increased the csDMARD dose. These two cases progressed without worsening arthritis.
CTLA4-Ig: cytotoxic T-lymphocyte antigen 4-immunoglobulin; JAKi: Janus kinase inhibitor; IL-6Ri: interleukin-6 receptor inhibitor;.TNFi: tumour necrosis factor-alpha inhibitor.

Supplementary Figure 4. Retention rates by drug formulation in the pulmonary non-tuberculous mycobacterial (PNTM) disease group
In the PNTM group, the 24-month retention rate was the highest at 75% for cytotoxic T-lymphocyte antigen 4-immunoglobulin (CTLA4-Ig) and IL-6Ris (log-rank test).
IL-6Ri: interleukin-6 receptor inhibitor; JAKi: Janus kinase inhibitor; TNFi: tumour necrosis factor-alpha inhibitor.

Supplementary Figure 5. Efficacy evaluation by the Clinical Disease Activity Index (CDAI) in the PNTM disease group
In the PNTM group, the proportions of patients with remission, low disease activity, moderate disease activity, or high disease activity based on the CDAI at treatment initiation and 24 months after treatment initiation were, respectively, as follows: 0%, 3.7%, 25.9%, and 70.4% at treatment initiation and 14.8%, 44.5%, 29.6%, and 11.1% at 24 months (Pearson's chi-square test).
HDA: high disease activity; LDA: low disease activity; MDA: moderate disease activity; PNTM: pulmonary non-tuberculous mycobacterial.
