# Supplementary figures and images for: CT informs detection and treatment options in rheumatoid arthritis complicated by pulmonary non-tuberculous mycobacterial disease from the FIRST registry
Source: RMD Open. 2024 Jun 12;10(2):e004049. doi: 10.1136/rmdopen-2023-004049 (PMC11177696; doi:10.1136/rmdopen-2023-004049)

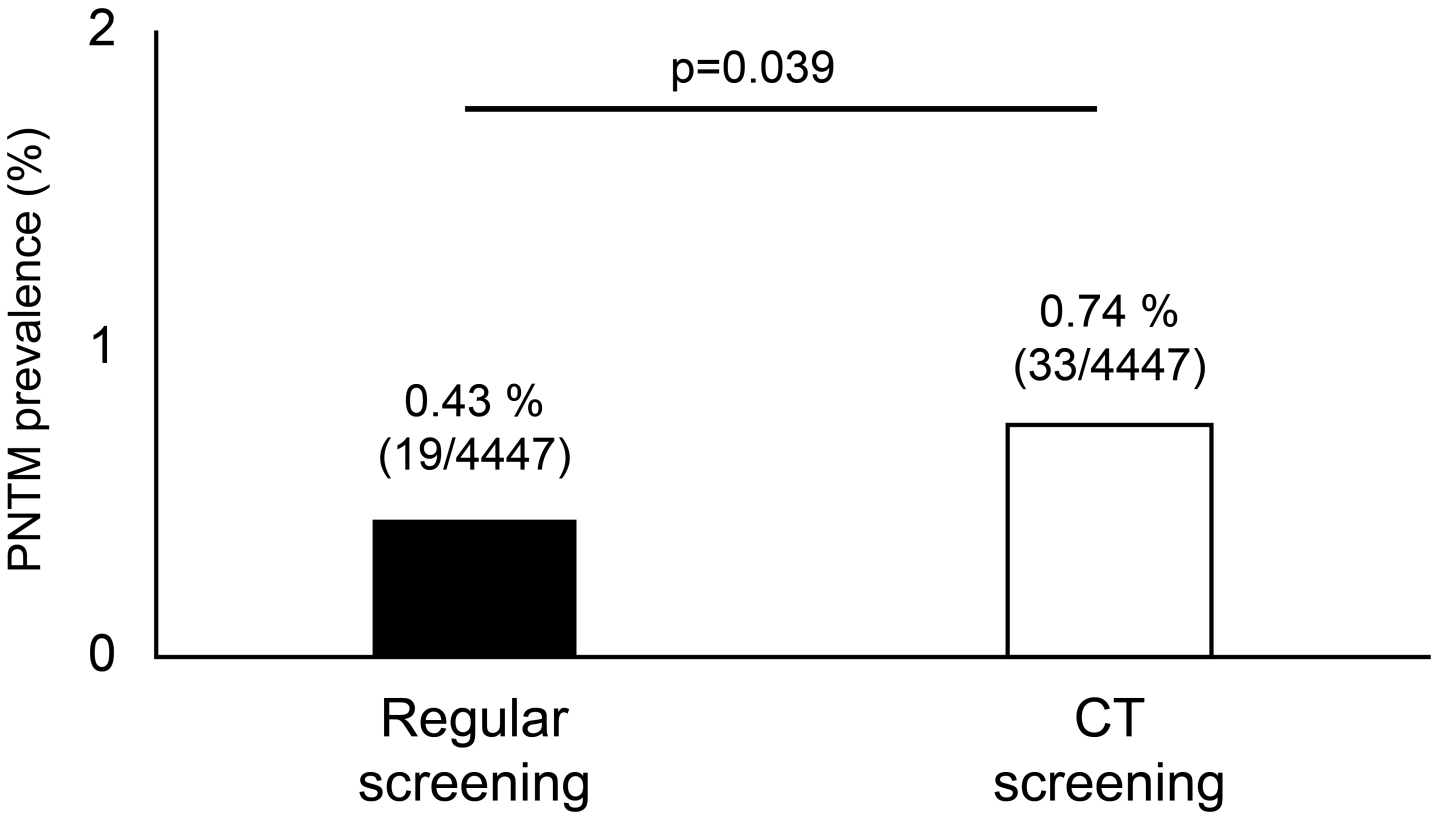

Supplement: Supplementary data [file rmdopen-2023-004049supp002.pdf]

Case 1

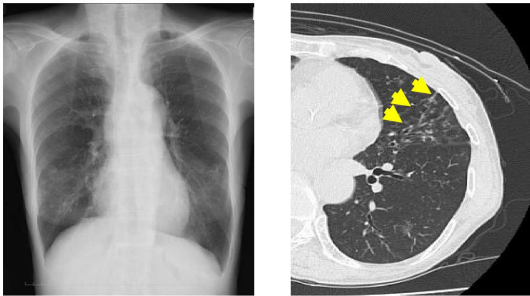

Case 2

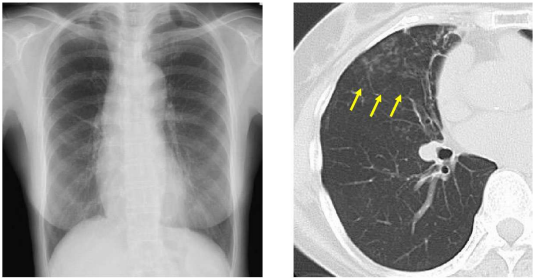

Case 3

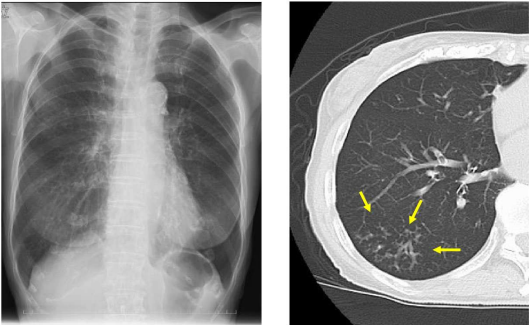

Case 4

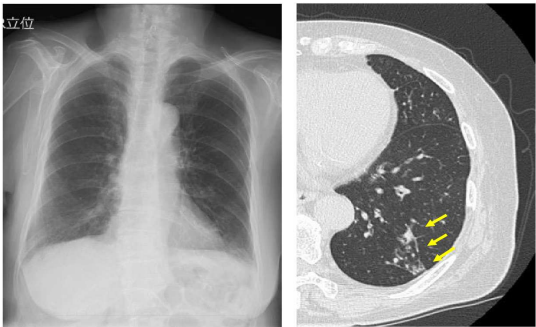

Case 6

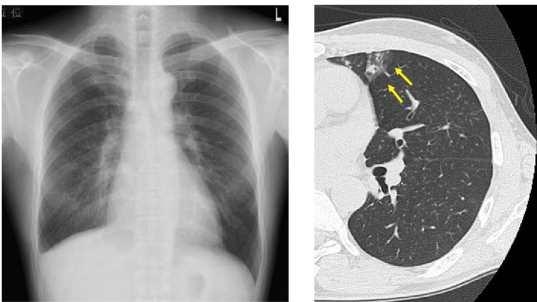

Case 7

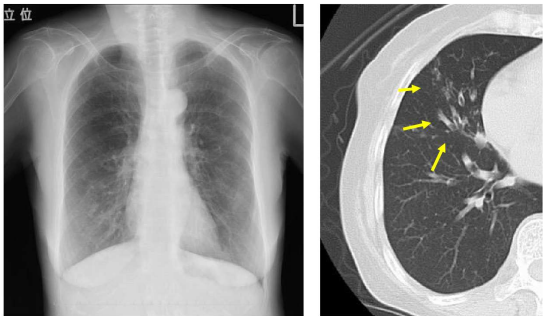

Supplement: Supplementary data [file rmdopen-2023-004049supp003.pdf]

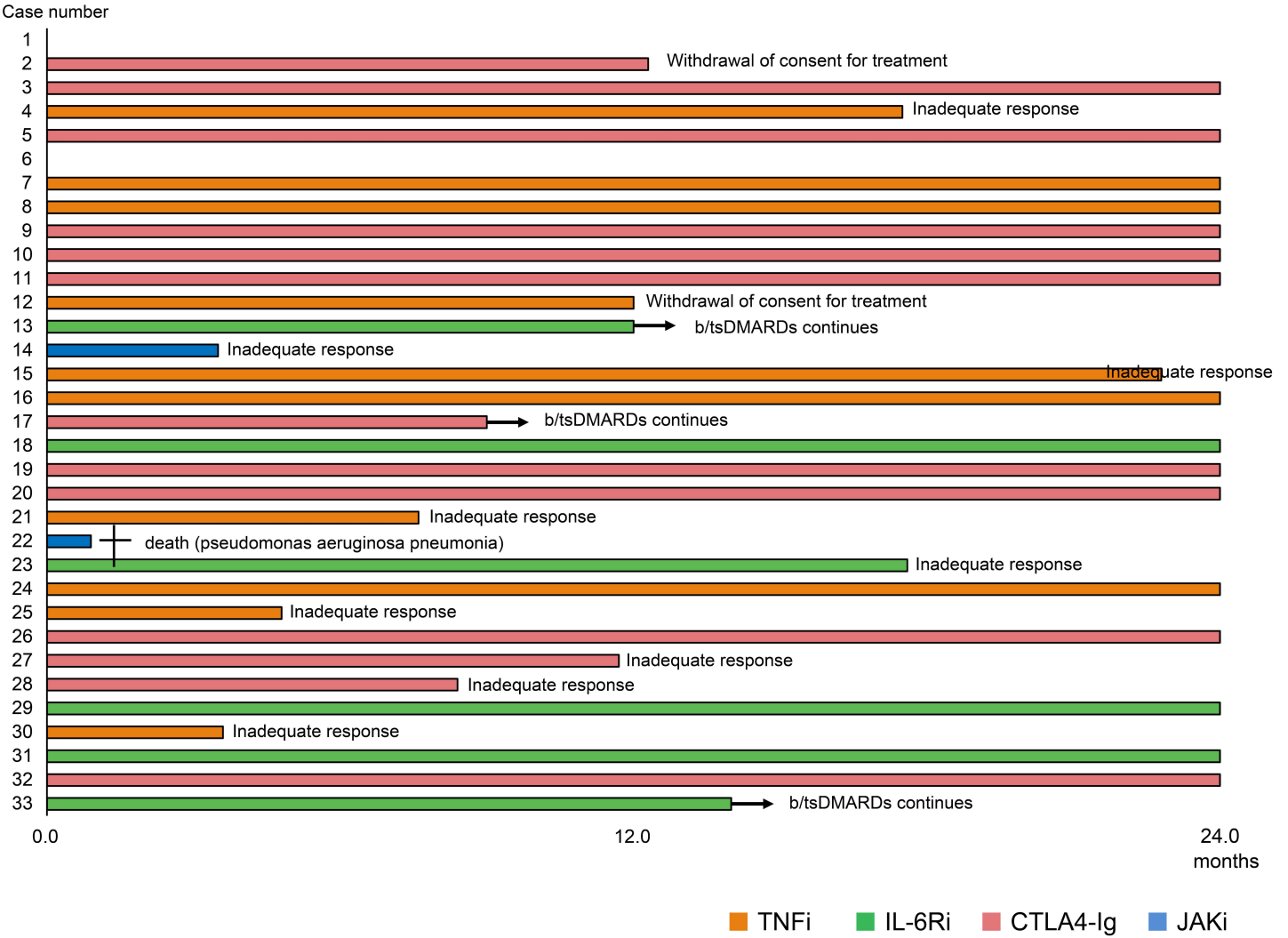

Supplement: Supplementary data [file rmdopen-2023-004049supp004.pdf]

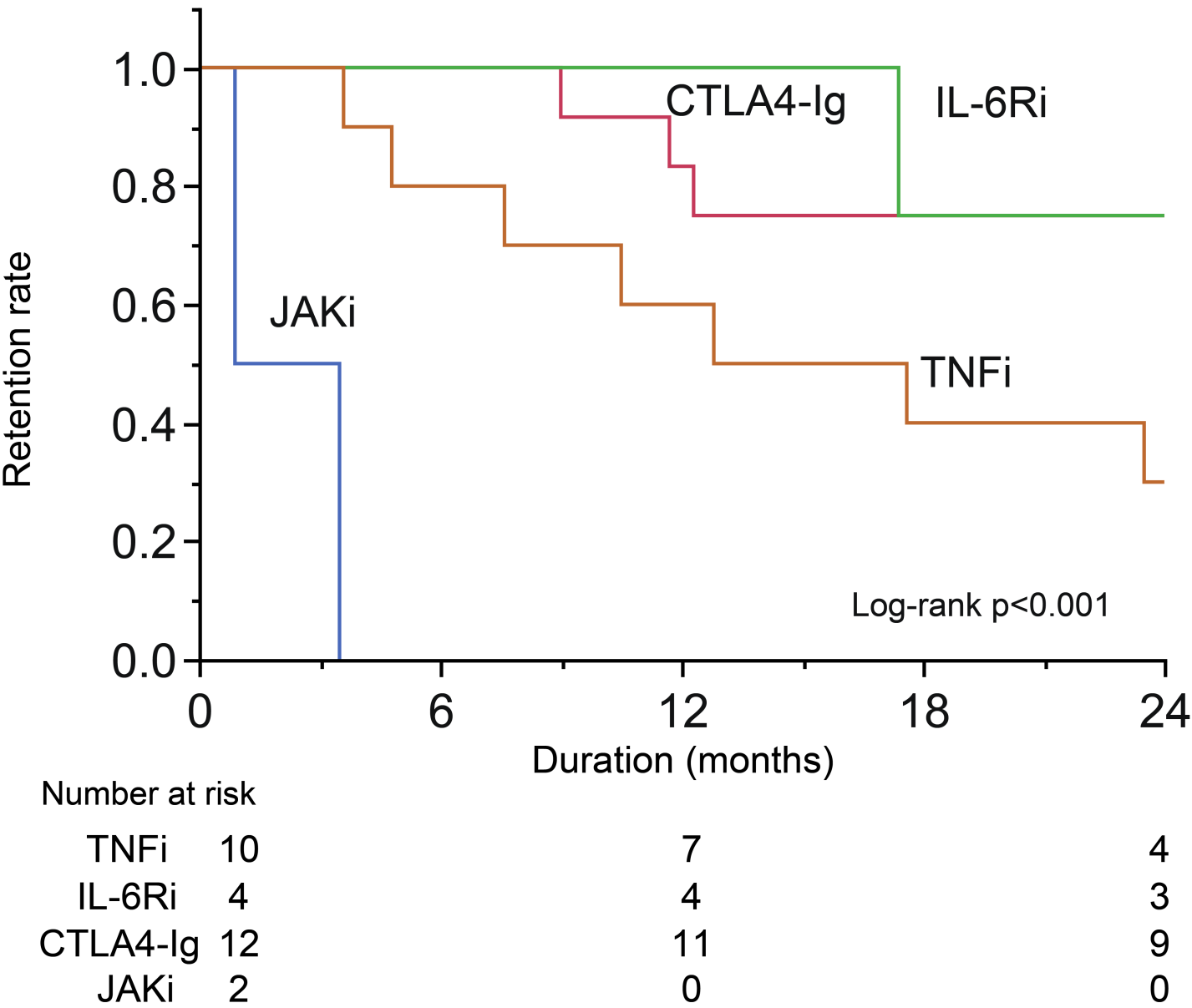

Supplement: Supplementary data [file rmdopen-2023-004049supp005.pdf]

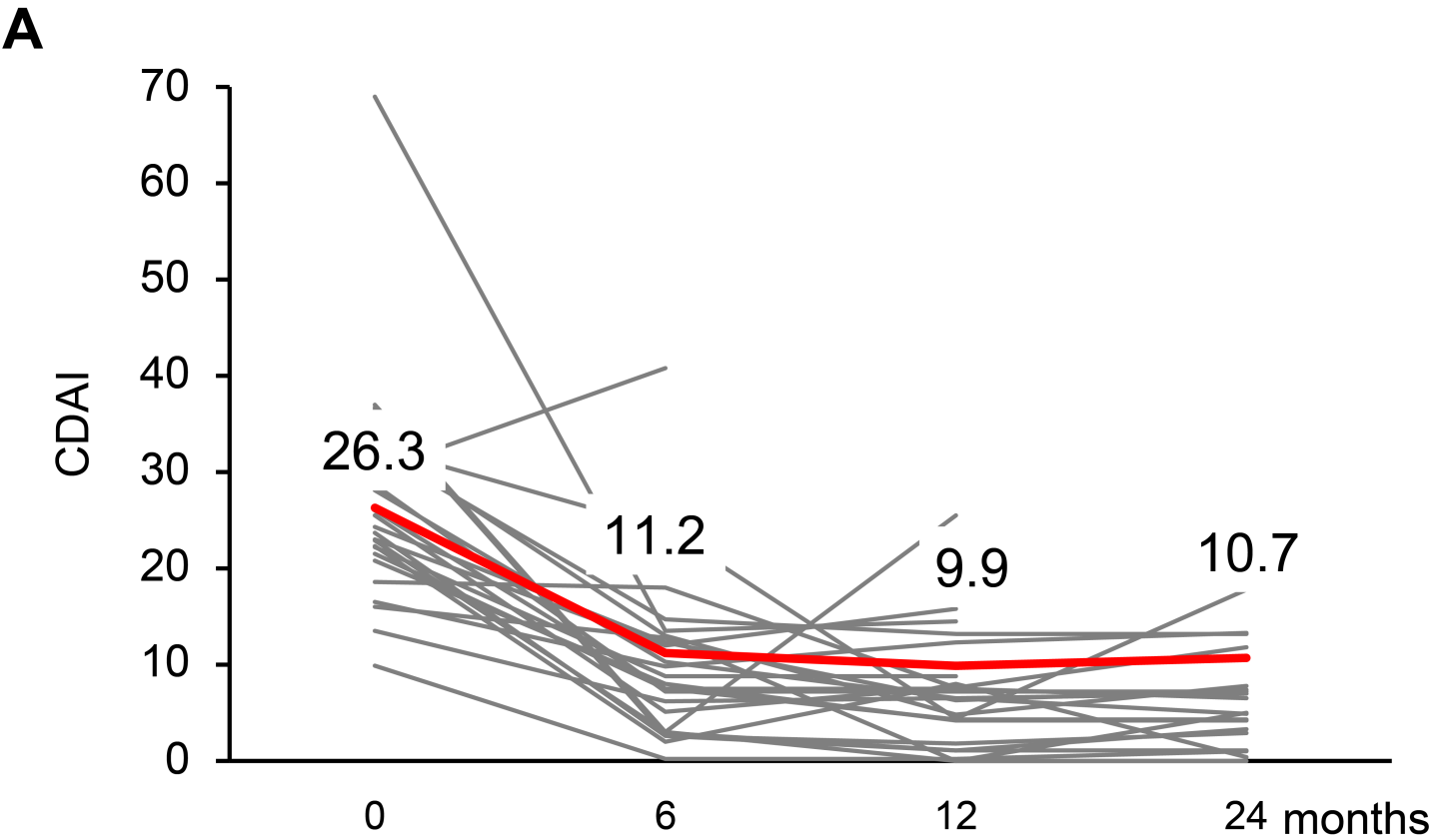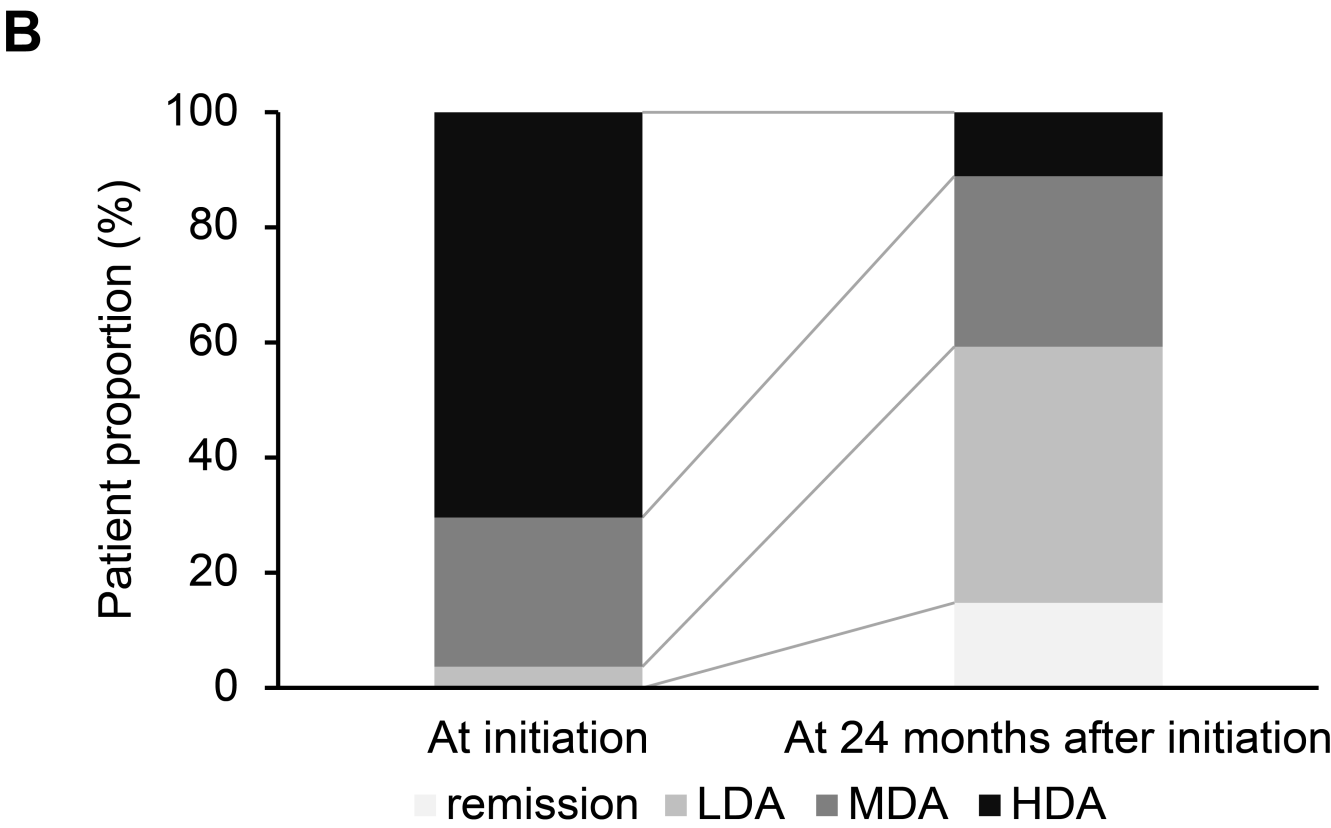

Supplement: Supplementary data [file rmdopen-2023-004049supp006.pdf]
